# Testing Conclusions From Functional Imaging of Working Memory with Data From Acute Stroke

**DOI:** 10.1155/2007/396946

**Published:** 2007-02-12

**Authors:** Lisa E. Philipose, Hannah Alphs, Vivek Prabhakaran, Argye E. Hillis

**Affiliations:** ^1^Department of Neurology, Johns Hopkins University School of Medicine, Baltimore, MDUSA; ^2^Department of Physical Medicine and Rehabilitation, Johns Hopkins University School of Medicine, Baltimore, MDUSA; ^3^Department of Radiology, Johns Hopkins University School of Medicine, Baltimore, MD,USA; ^4^Department of Cognitive Science, Johns Hopkins University School of Medicine,Baltimore, MDUSA

**Keywords:** Working memory, acute stroke, magnetic resonance perfusion-weighted imaging, diffusion-weighted imaging

## Abstract

Functional imaging studies indicate that the left hemisphere mediates verbal working memory, while the right hemisphere mediates both verbal and spatial working memory. We evaluated acute stroke patients with working memory tests and imaging to identify whether unilateral dysfunction causes deficits in spatial and/or verbal working memory deficits. While left cortical stroke patients had verbal working memory impairments (*p* < 0.003), right cortical stroke patients had both verbal (*p* < 0.007) and spatial working memory (*p* < 0.03) impairments, confirming functional imaging results. Patients with transient ischemic stroke and patients with non-cortical stroke did not have significant deficits in working memory in either modality.

## 1. Background

Functional imaging studies identify brain areas where a hemodynamic response correlates with a particular event (stimulus or response). Such results have been interpreted as revealing areas of neural activation associated with various cognitive processes. However, functional imaging evidence is strictly correlational; it shows regions that are *engaged* in an event. To confirm that "activated" neural regions are *essential* to the cognitive function, one can demonstrate that damage to those regions disrupts the associated cognitive function [2,8]. Most lesion studies have studied patients months or years after stroke, after substantial reorganization of structure/function relationships. Therefore, it is impossible to determine whether or not the lesion initially disrupted the function. Studies of acute stroke are complicated by the fact that both structural damage and areas of low blood flow may contribute to observed deficits [23,25]. Diffusion-weighted imaging (DWI, which shows dense ischemia) and perfusion-weighted imaging (PWI, which shows areas of hypoperfusion), can identify dysfunctional brain tissue acutely after stroke, before reorganization of structure/functional relationships [1,9]. Therefore, DWI and PWI along with cognitive assessment in acute stroke can be used to confirm that neural dysfunction in a region causes the deficit predicted by functional imaging.

We illustrate this approach to studying brain/behavior relationships by testing conclusions from functional imaging studies of working memory (WM). Working memory is the capacity to hold information for short periods of time. Traditionally, two distinct forms of working memory, verbal and spatial, have been found

ISSN 0953-4180/07/$17.00 © 2007 – IOS Press and the authors. All rights reserved to be asymmetrically represented in the left and right hemispheres, respectively [10,11,16,24]. Recent functional magnetic resonance imaging (fMRI) studies indicate that the left hemisphere mediates verbal WM, while the right hemisphere is involved in *both* spatial and verbal WM [5,15,17,20]. We aimed to evaluate this conclusion by testing the hypothesis that acute right hemisphere dysfunction causes impaired spatial and verbal WM, while acute left hemisphere dysfunction causes impaired verbal WM only. While there are more complex relationships between particular brain regions and particular types of WM (e.g. semantic versus phonological verbal WM) that have been revealed by various functional imaging studies, this hypothesis was the simplest, most reliable, and most feasible to test in a study of acute stroke patients enrolled in the course of a year at one hospital.

## 2. Methods

A consecutive series of consenting right-handed patients with acute ischemic stroke were studied within three days of symptom-onset. Patients were studied within 24 hours of hospital admission. Most (75%) patients were studied within 24 hours of symptom onset; however, 25% were studied within two to three days because they presented to the hospital more than a day after their symptoms began. We decided to include such patients to increase participation in the study. This time window limited the degree of reorganization – the extent to which other brain areas may have assumed functions of injured brain. Additionally, consenting right – handed patients with transient ischemic attack (TIA; defined as resolution symptoms within 24 hours and stroke excluded by MRI) were enrolled to serve as hospitalized controls with similar ages and other demographic factors, vascular risk factors, and stressors of hospitalization to those of the stroke patients. It was necessary to enroll these controls, to confirm that deficits observed in stroke patients were due to the stroke and not due to normal aging, vascular disease or risk factors, or stressors associated with hospitalization. Additional exclusion criteria included: (1) premorbid dementia or other neurological or psychiatric disease; (2) contraindication for MRI; (3) diminished level of consciousness; (4) lack of premorbid proficiency in English; (5) visual field deficits (by medical records and bedside examination); (6) motor deficits precluding performance of computer tasks; (7) language deficits that interfered with comprehension of the task; or 8) left neglect, defined by $>$10% errors on the left half of the page in line cancellation. All patients had the following MRI sequences as part of the Johns Hopkins Acute Stroke Protocol: diffusion weighted imaging (DWI) and perfusion weighted imaging (PWI), axial T2, fluid attenuated inversion recovery (FLAIR), gradient echo (GE), and apparent diffusion coefficient (ADC) maps. The reported analyses used DWI (after confirming the acuity of the lesion as dark on ADC maps) and PWI (coregistered to T2 to provide anatomical boundaries that are less visible on PWI). Whole brain coverage was obtained by DWI and PWI scans of 5 mm thick slices. Volumes of tissue dysfunction on DWI and/or PWI were calculated using the Image J program. If a patient had imaging abnormalities on both DWI and PWI, the larger calculated volume was used. Volume of PWI abnormality was measured on time to peak maps, defining hypoperfused tissue as voxels where there was $>$4 seconds delay in time to peak arrival of contrast, relative to the homologous region in the normal hemisphere.

Stroke was defined as acute infarct on DWI and/or hypoperfusion on PWI with corresponding neurological deficits. Stroke patients were further categorized as cortical strokes (right or left) or noncortical strokes (basal ganglia, thalamus, internal capsule, white matter only, brain stem, or cerebellar). TIA was defined as normal DWI/PWI and resolution of neurological deficits within 24 hours.

Participants, all right-handed, performed subject-paced computerized verbal and spatial WM tasks with their dominant hand, unless they had right arm or hand weakness such that they preferred to use the left, nondominant-hand. On each of 48 trials, they (1) encoded 3, 4, 5, or 6 to-be-remembered letters or locations, (2) maintained these letters (or locations) across a 5-second unfilled delay, and (3) indicated whether or not a single probe letter (or location) was part of the memory set. For the verbal task, the to-be-remembered letters and probe letters were shown in different cases, so that the subjects encoded the verbal identity of letters rather than their visual appearance. They also completed span tasks, which can be performed by patients with even moderate aphasia [24]. The digit span task required subjects to immediately repeat random sequences of digits increasing by one digit in length. The test was stopped after two consecutive incorrect responses, and the previous span length was recorded as the subject's span. Similarly, the spatial span task required subjects to tap random sequences (of increasing length) of blocks on a page immediately following the examiner's tapping. Participants were required to perform the span tasks in forward and backward sequence relative to the examiner, and a total score summing the performance length in each was noted. Not all study participants performed all tasks due to time constraints, fatigue, or other required medical care/procedure which prevented completion. If a participant failed to complete all 48 trials of a WM task or failed to complete the span task as described above, all of the person's data for that incomplete task was discarded.

WM involves three components including the ability to (1) encode information, (2) accurately maintain this information, and (3) retrieve the information. Thus, WM deficits were defined as more than two standard deviations below the mean composite score of these three components (sum of deviations as determined by z-scores in accuracy, encoding time, and response time) of 20 normal, healthy volunteers recruited by advertisement for a separate study of WM. Span deficits were defined as more than two standard deviations below the published age/education-adjusted normal mean [26]. We evaluated associations between frequencies of deficits and areas of hypoperfusion/infarct using chi-squared tests. The study was approved by the Johns Hopkins Institutional Review Board.

## 3. Results

A total of 67 patients, 40 with stroke and 27 with TIA were enrolled. Age ranged from 32–82 years (mean 57.5 $\pm$ 12.0) for acute stroke patients and 35–85 years (59.8 $\pm$ 13.5) for TIA patients. Education ranged from 12–16 years (mean 15.6 $\pm$ 1.2) for acute stroke, 9–18 years (13.3 $\pm$ 3.0) for TIA patients.

To determine if stroke impaired verbal or spatial WM, we compared performance of stroke patients to TIA patients (Fig. 1). A higher proportion of stroke patients showed deficits in spatial span ($\chi^2 = 5.1$, $df = 1, p < 0.03$) and verbal WM ($\chi^2 = 12.3, df = 1$, $p < 0.0005$). There was no difference in digit span or spatial WM for stroke versus TIA patients.

To distinguish the selective impact of cortical dysfunction, we categorized strokes as cortical or noncortical (basal ganglia, thalamus, internal capsule, white matter only, brain stem, or cerebellar) (Fig. 2). A higher proportion of cortical stroke patients than TIA patients showed deficits in spatial span ($\chi^2 = 9.9$, $df = 1$, $p < 0.002$), spatial WM ($\chi^2 = 4.2$, $df = 1$, $p < 0.05$) and verbal WM ($\chi^2 = 11.5$, $df = 1$, $p < 0.0008$), while no significant differences were found between noncortical stroke and TIA patients. A higher proportion of cortical stroke patients than noncortical stroke patients showed deficits in spatial span ($\chi^2 = 7.1$, $df = 1, p < 0.008$) and spatial WM ($\chi^2 = 4.7, df = 1$, $p < 0.03$). Again, no difference in digit span performance was noted.

To determine relative contributions of each hemisphere in verbal and spatial WM performance, we substratified cortical strokes as left or right cortical (Fig. 3). A higher proportion of right cortical stroke patients than TIA patients had deficits in spatial span ($\chi^2 = 13.4$, $df = 1$, $p < 0.0004$) and spatial WM ($\chi^2 = 5.2$, $p < 0.03$). There was no difference between left cortical and TIA patients on spatial tasks. In contrast, both right cortical ($\chi^2 = 7.4$, $df = 1$, $p < 0.007$) and left cortical stroke ($\chi^2 = 8.9$, $df = 1$, $p < 0.003$) patients more commonly had deficits in verbal WM than TIA patients. Again, there was no difference in digit span. To determine whether a greater volume of stroke in right cortical stroke patients may have contributed to these results, we compared the volume of tissue dysfunction on DWI/PWI in left versus right cortical strokes. The volume of dysfunctional tissue was not significantly different, mean 20.7 $cm^3 \pm$ 18.0 for left cortical strokes and mean 36.1 $cm^3 \pm$ 45.3 for right cortical strokes ($t = 1.12$, $df = 16$, $p = 0.28$). We attempted to further subcategorize patients by the lobe(s) involved as depicted in Table 1; however, there were too few patients with infarct involving each lobe or combination of lobes to determine any association between lobe(s) involved and WM performance. It should be noted that 56% of the patients with cortical infarct had lesions that included at least part of the frontal lobe.

We also attempted to analyze inter-group performance measures for the four working memory tasks used in this study as shown in Table 2. We were interested in determining whether forward or backward performance of span tasks affected performance on the verbal or spatial WM tasks. We were also interested in analyzing inter-group differences in the three individual components of our pooled WM score: 1) encoding time (the self-paced time a patient used to view/encode a "to-be-remembered" WM stimulus), 2) reaction time (the self-paced time a patient used to decide on whether a probe stimulus matched the initially presented stimulus), and 3) accuracy rate. However, due to large intragroup variability, we were not able to identify any significant relationships between side of lesion and any of the three components.

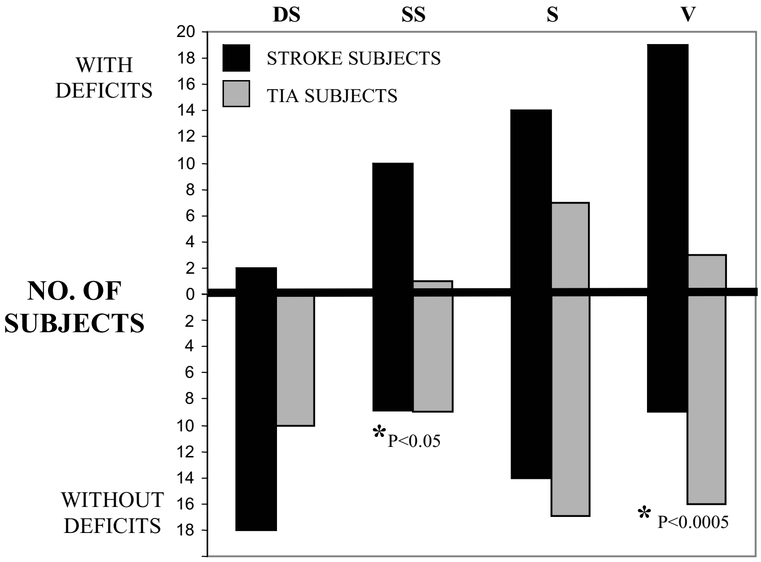

Fig. 1. **Stroke subjects were more impaired than TIA subjects in working memory performance** DS = Digit span task, SS = Spatial span task, S = Spatial WM task, V = Verbal WM task.

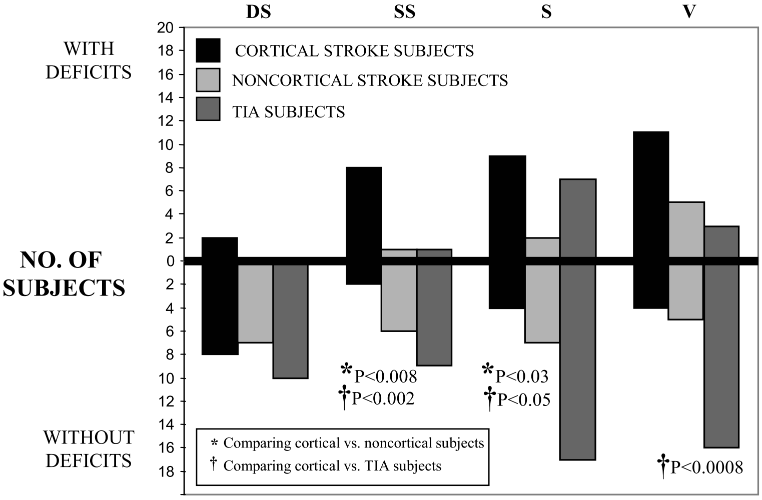

Fig. 2. **Cortical stroke subjects were more impaired in working memory performance than noncortical or TIA subjects** DS = Digit span task, SS = Spatial span task, S = Spatial WM task, V = Verbal WM task.

## 4. Discussion

Overall, a higher proportion of stroke patients than TIA patients showed verbal and spatial WM deficits, confirming that hypoperfusion/infarct can impair performance in these cognitive tasks. A higher proportion of patients with cortical than noncortical stroke showed deficits in both WM tasks and spatial span, suggesting that cortical regions play a greater role than noncortical regions in spatial and verbal WM and spatial span, consistent with data from functional imaging [5,16,17, 24].

While right cortex was found to be important for performance in spatial WM and spatial span, both right and left cortex were found to be important for performance in verbal WM. While left cortical stroke patients were compromised in only verbal WM, right cortical stroke patients were compromised in both verbal and spatial WM. This converges with fMRI results, indicating an essential role for right cortex in multimodality or modality-independent WM [5,17]. However, it should

Table 2
Mean (standard deviation) accuracy and reaction times for participants in working memory tasks

| | | **All Strokes** | **Cortical** | **Noncortical** | **L cortical** | **R cortical** | **TIA** |
|---|---|---|---|---|---|---|---|
| **Digit Span** | Forward span | 7.9 (2.3) | 7.1 (2.5) | 8.9 (2.2) | 9.3 (1.6) | 6.1 (2.3) | 10.3 (2.2) |
| | Backward span | 3.4 (1.7) | 2.7 (1.3) | 4 (1.7) | 3.7 (0.6) | 2.3 (1.4) | 5.8 (2.6) |
| | Total span | 11.3 (3.6) | 9.8 (3.6) | 12.9 (3.3) | 13 (1.0) | 8.4 (3.4) | 15.9 (3.5) |
| **Spatial Span** | Forward span | 5.2 (2.4) | 4 (2.4) | 6.7 (1.6) | 5.7 (2.9) | 3.3 (2.0) | 6.5 (1.1) |
| | Backward span | 4.2 (3.4) | 3 (3.6) | 5.9 (3.2) | 7 (4.6) | 1.3 (0.8) | 4.6 (1.7) |
| | Total span | 9.3 (5.4) | 7 (5.6) | 12.6 (4.1) | 12.7 (7.4) | 4.6 (2.4) | 11.1 (2.4) |
| **Verbal WM** | Encoding Time in msec | 9646.4 (7117) | 7203 (2520) | 9203.3 (4248) | 8184.2 (2577) | 6081.8 (2080) | 5463.3 (2587) |
| | Retrieval Time in msec | 4571 (4143) | 3668.1 (1583) | 4200.4 (3801) | 3223 (1003) | 4176.8 (2026) | 2339.9 (734) |
| | %Accuracy | 0.71 (0.16) | 0.65 (0.23) | 0.45 (0.39) | 0.67 (0.19) | 0.71 (0.14) | 0.86 (0.13) |
| **Spatial WM** | Encoding Time in msec | 7146.9 (4719) | 6541 (2822) | 6492.1 (4830) | 5784.5 (1893) | 7014.5 (3307) | 5618.5 (2562) |
| | Retrieval Time in msec | 6116.6 (5429) | 6230 (3786) | 5788.6 (7301) | 4972.2 (1950) | 7016.1 (4535) | 4530.3 (2623) |
| | % Accuracy | 0.69 (0.15) | 0.66 (0.14) | 0.76 (0.14) | 0.71 (0.11) | 0.63 (0.15) | 0.69 (0.16) |

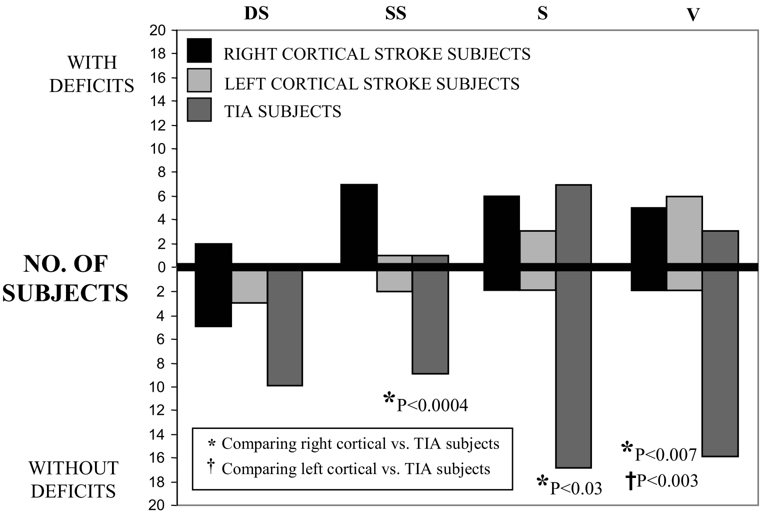

Fig. 3. **Left cortical stroke subjects were impaired in verbal working memory, while right cortical stroke subjects are impaired in both verbal and spatial working memory** DS = Digit span task, SS = Spatial span task, S = Spatial WM task, V = Verbal WM task.

be noted that some studies have found left hemisphere activation during spatial working memory tasks [12, 22]. Although not the case here, other studies have also found interhemispheric interaction where dysfunction in the dominant (left) hemisphere may be associated with impairment of functions in that hemisphere and with improvement of functions in the nondominant (right) hemisphere [18].

Several factors should be further investigated. First, hypoperfusion/infarct should be localized to smaller regions, to evaluate the impact of focal neural dysfunction on WM performance. In particular, fMRI studies indicate a specific role of dorsolateral prefrontal cortex (DLPFC) in WM [3,5,7,21]. If this area is essential for WM, lesions in DLPFC should cause impaired WM. Sample size must be increased to strengthen statistical power to examine associations between dysfunctional brain regions and WM deficits. Also, the effect of volume of infarct (on DWI) and hypoperfusion (on PWI) should be evaluated. Ideally, groups of left cortical and right cortical stroke patients should be matched for involvement of the DLPFC, volume of infarct, and volume of hypoperfusion in this region. Alternatively, multivariate regression analysis can be used to evaluate the effect of presence or absence of damage in various regions of interest, controlling for volume of infarct and hypoperfusion, on working memory performance. To account for the potential of brain reorganization even in acute stroke studies, we can also consider complementary data from transcranial magnetic stimulation (TMS) where temporarily interfering with the functioning of specific brain regions such as the prefrontal cortex, parietal cortex, and cerebellum have been found to be associated with impairments in components of working memory [4,13,19], see [14] for review. Further investigation is also required to explain the lack

Table 1
Percentage of patients in each group whose lesions involved each lobe(s) or noncortical region(s)

| | Right cortical | Left cortical |
|---|---|---|
| **Frontal** | 26.67% | 18.18% |
| **Parietal** | 13.33% | 36.36% |
| **Temporal** | 0.00% | 0.00% |
| **Occipital** | 6.67% | 9.09% |
| **Frontal-parietal** | 20.00% | 18.18% |
| **Temporal-parietal** | 6.67% | 18.18% |
| **Frontal-temporal-parietal** | 20.00% | 0.00% |
| **Occipital-temporal-parietal** | 6.67% | 0.00% |
| **TOTAL** | **100%** | **100%** |
| | **Right noncortical** | **Left noncortical** |
| **Basal ganglia** | 40.00% | 28.57% |
| **Thalamus** | 10.00% | 14.29% |
| **Internal capsule** | 10.00% | 0.00% |
| **White matter only** | 0.00% | 14.29% |
| **Brainstem** | 10.00% | 28.57% |
| **Cerebellum** | 30.00% | 14.29% |
| **TOTAL** | **100%** | **100%** |

of difference in performance between stroke and TIA subjects in the digit span task. However, there is similar evidence that digit span is preserved in patients with prefrontal lesions and may be relatively insensitive to cortical damage [6].

In summary, new insights into brain regions responsible for span and WM tasks from this study are limited. However, the results illustrate how behavioral and imaging data in acute stroke patients can predict whether or not areas of "activation" associated with a specific task in functional imaging are essential for that task. Evidence from this investigation confirmed a hypothesis generated from fMRI studies that right cortical regions play a critical role in both spatial and verbal WM and spatial span, while left cortical regions play a critical role only in verbal WM.

## Acknowledgments

The research reported in this paper was supported by NIH R01: NS047691 and P41 RR15241 and DC05375.
